# Determinants of Student Loyalty in Higher Education: A Structural Equation Approach for the Bucharest University of Economic Studies, Romania

**DOI:** 10.3390/ijerph19095527

**Published:** 2022-05-02

**Authors:** Steluta Todea, Adriana AnaMaria Davidescu, Nicolae Al. Pop, Tanase Stamule

**Affiliations:** 1 School of Marketing, The Bucharest University of Economic Studies, 010374 Bucharest, Romania; todeasteluta09@stud.ase.ro; 2 Department of Statistics and Econometrics, The Bucharest University of Economic Studies, 010374 Bucharest, Romania; 3 Department of Education, Training and Labour Market, National Scientific Research Institute for Labour and Social Protection, 010374 Bucharest, Romania; 4 Faculty of Marketing, The Bucharest University of Economic Studies, 010374 Bucharest, Romania; nicolae_al_pop@yahoo.com; 5 Department of Business Administration, The Bucharest University of Economic Studies, 010374 Bucharest, Romania; tanase.stamule@fabiz.ase.ro

**Keywords:** student loyalty, satisfaction, trust, commitment, perceived quality, brand associations, higher education, relationship marketing, brand marketing

## Abstract

In today’s world, the higher education system represents a means of developing the national and global economy by providing individual and collective benefits. Student loyalty is a critical measure in the success of higher education institutions that aim at retaining students until graduation and then attracting them back. As they fuel the knowledge economy, universities create a more educated labor market, providing employment and higher salaries. Having this major significance, it is vital to study the key determinants that persuade stakeholders to form long-term relationships with universities, revealing high loyalty. Aiming to stay competitive and survive the drastic, ongoing changes, successful universities thrive on building loyalty among all stakeholder groups, especially students. Despite the importance of the higher education institutions (HEIs), little research has been conducted on student loyalty. Therefore, the main objectives of this study are to empirically examine the key factors influencing student loyalty by testing two models, namely perceived quality, brand associations, satisfaction, trust, and commitment, and to test the relationships among them. To analyze the data, a confirmatory factor analysis was applied where it explored the associations between items and constructs and, then, utilized a structural equation model (SEM) to investigate the relationships existing between constructs with the application of the STATA program. A structured questionnaire comprising of 66 questions was developed, using a five Likert scale. A total of 682 students from the Bucharest University of Economic Studies filled in the questionnaire. In both cases, the findings reveal that commitment has the most significant direct impact on loyalty. The other factors have an indirect effect, satisfaction having the most significant total effect, followed by trust and commitment. Therefore, universities must focus on improving service quality to develop positive brand associations, student satisfaction, trust, and commitment in developing student loyalty.

## 1. Introduction

Loyalty is one of the most important character traits everyone should have. In all areas of life, whether personal or professional, loyalty is priceless and a top priority in every kind of relationship.

In today’s globalized world, the higher education system has to be seen as a service industry and universities as service providers. Therefore, universities should focus on the needs and wants of all stakeholder groups, especially on those of students. Moreover, to be successful, the higher education system must be directly linked to the increasingly complex requirements of profit [1]. Additionally, major changes and fierce competition in the educational market force universities to turn their attention to brand marketing, to differentiate their educational offerings from those of their competitors. Brand loyalty is a primary measure of effective brand marketing and a partial measure of brand equity [1,2,3], implying complex relationships between the customer and a particular brand [4,5]. It might be viewed as the most crucial aspect of relationship marketing, revealing a psychological attachment to the brand [6]. Brand loyalty represents the core of the marketing activities, being a key to integrated marketing [7]. Most studies on brand loyalty have focused on the measurement of antecedents and mediating roles of different variables, such as service quality, satisfaction, trust, and commitment [8,9,10,11,12,13,14,15].

Applying marketing concepts to higher education is shared, from academic branding, [16,17,18,19,20], measuring brand equity [21,22,23,24] to relationship marketing [25,26]. Regarding educational services, customer brand loyalty requires the development of solid relationships with students, who economically support the existence of the university activities [27], even after graduation [28]. Aiming to retain students until graduation and then attract them back [29], student loyalty supports university administrators in developing suitable programs that initiate, build, and maintain successful long-term relationships with students [30].

The purpose of the present study involves the empirical testing of a conceptual model of student loyalty. As in other industries, higher education studies on student loyalty focus mainly on developing conceptual frameworks that integrate the antecedents and mediators of loyalty. For instance, Chandra et al. [31] revealed a significant positive influence of service quality towards student satisfaction and student satisfaction on student loyalty. Annamdevula and Bellamkonda [30], testing a mediation model that links service quality and student loyalty via student satisfaction, proved the mediator role of student satisfaction between the two constructs. Kunanusorn and Puttawong [32] showed that student satisfaction, trust, perceived value, and university image positively influence student loyalty. Furthermore, Purgailis and Zaksa [33] demonstrated that student perceived quality correlates with academic staff, study content, readiness for the labor market, and acquired skills, which consequently influence student loyalty, and Perin et al. [34] found out that trust has a positive impact on students’ commitment and loyalty, and commitment has a positive effect on students’ loyalty.

Moreover, Carvalho and Mota [28] demonstrated that trust in faculty and staff, including in management, increases students’ perception of the value of the university, which in turn affects student loyalty. Furthermore, Rojas-Méndez et al. [29] revealed that perceived service quality and student satisfaction translate indirectly into student loyalty through the mediation of trust and commitment. Finally, Helgesen and Nesset [35] showed that student perception of the university’s’ reputation positively influences student loyalty. Moreover, reputation directly affects loyalty, while students’ satisfaction positively influences both student loyalty and the universities’ reputation.

**Figure 1:**
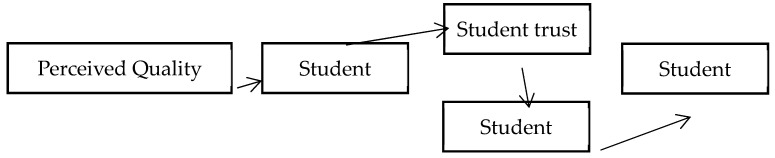
Model processed after Rojas-Méndez et al. [29]. “Reprinted/adapted with permission from Ref. [29].

The main objectives of the present study are to empirically test and identify the key factors affecting student loyalty based on two models with structural equations, developed based on research conducted by Rojas-Méndez et al. [29](Figure 1.)

Based on the above model, two models have been tested in this paper, namely:

1. perceived quality ≥ satisfaction ≥ trust ≥ commitment to the brand ≥ brand loyalty;
2. brand associations ≥ satisfaction ≥ trust ≥ commitment to brand ≥ loyalty to brand.

The paper brings its contributions to the literature in several ways. In the first way, the determinants of student loyalty have been studied before, but the majority of the studies have covered just main determinants of loyalty such as student satisfaction and ignored perceived quality and brand associations. This study has been conducted to fill these knowledge gaps and propose a comprehensive model depicting elaborate relationships of all important antecedents of student loyalty. Thus, starting from Rojas-Méndez et al.’s [29] model, the paper proposes two alternative models, incorporating this time the impact of perceived quality and brand associations. In the second way, the paper empirically tests both of these models on the students from Bucharest University of Economic Studies. In the third way, the study successfully applies an SEM to identify the relationship among constructs. Thus, this research has hopefully opened up avenues for other researchers to carry out such behavioral studies with larger sample sizes by applying STATA software with SEM analysis.

The paper is structured as follows. The second section is dedicated to a literature review and hypotheses development, while the third section is dedicated to research design and methodology. The next two sections are dedicated to empirical results and discussions, and the paper ends with the conclusions, main limitations, and future direction of research.

## 2. Materials and Methods

Recognizing the importance of loyalty in the marketing literature, practitioners and researchers started to study this concept a long time ago [36], trying to separate brand loyalty from customer loyalty, even though the two concepts are similar, and to identify the key factors that form loyalty to a company’s products and services [37]. Gaining customer loyalty is one of the most important goals of marketing [38,39]. Therefore, the success of any organization depends primarily on its capacity to attract, retain, and make consumers loyal to the brand [40,41,42]. Attracting new consumers is a self-evident goal, but retaining the existing ones is the real challenge for organizations. Loyal customers to the brand will always increase sales and customer share [43,44,45,46], being particularly important for long-term profitability [7,47,48,49,50] and reducing marketing costs [37,51,52]. Moreover, customer brand loyalty leads to sustainable competitive advantage [45,53,54]. The main difference between these two loyalty types is that customer loyalty revolves around customer spending, and brand loyalty is all about customer perception. The present study refers to both types because it aims to measure both student perception about the university and students spending different resources with the university.

The study and approach of customer brand loyalty should always start with a coherent and comprehensive definition and a thoroughly developed measure. Brand loyalty has had a long and dynamic past. Copeland [55] was the first who mentioned the idea of loyalty, followed by a large number of authors who provided various definitions see [56], which reveal the importance of this concept to marketing theory [4]. However, today, the concept lacks a generally accepted definition and a coherent and concrete measurement scale. Lovelock and Wirtz ([57], p. 629) describe brand loyalty as “a customer’s commitment to continue patronizing a specific firm over an extended period.” The most cited definitions in the literature are those provided by Oliver (1999) and Jacoby and Chestnut [56]. Oliver ([58], p. 392) defined loyalty as “*a deeply held commitment to rebuy or re-patronize a preferred product or service consistently in the future, despite situational influences and marketing efforts having the potential to cause switching behavior.*” Jacoby and Chestnut [56] suggested that brand loyalty should be viewed as a biased behavioral response, expressed over a while, starting from a decision and considering one or more alternative brands, being a function of psychological processes. Another important definition of brand loyalty in the marketing literature is provided by Aaker ([51], p. 39), who defines brand loyalty as the measure of attachment that a consumer has to a brand. In his view, brand loyalty mirrors the likelihood of a customer changing the brand when there are changes in price or product features [51].

### 2.1. Student Loyalty

Student loyalty refers to students’ loyalty to the university expressed after the educational experience [35,59,60]. In addition, it refers to student willingness to promote, praise, and recommend the institution to family, friends, and other people known from different backgrounds whenever opportunities come [32]. The success of an educational institution depends both upon the loyalty of formal attendees and former students. As a result, student loyalty implies loyalty both during and after the student’s educational experience with the university (27).

### 2.2. Perceived Quality

Perceived quality can be defined as the customer’s perception of the overall quality or superiority of a product or service concerning the proposed purpose and existing alternatives [51,61]. The quality of products or services influences market share and customer satisfaction [62]. According to Aaker [51], perceived quality gives value to a brand in several ways. Namely, high quality is an excellent reason for the consumer to buy a brand and allows it to differentiate itself from its competitors, charge a premium price, and have a solid base for brand extension. The quality of products or services is often conceptualized as a comparison between performance expectations and actual perceptions of performance [61]. In the educational field, perceived quality refers to the students’ perception of the overall quality regarding the academic service. Good service quality improves customer satisfaction and causes long-term benefits in profitability and market share [63].

### 2.3. Brand Associations

Brand associations are closely related to the information held by the customer about the brand, whether positive or negative [64]. These are, in fact, the benefits and attributes of some products or services through which the brand can be competitive and compared with other brands [65]. The more associations a product generates, the more it will be stored in the consumer’s mind, developing brand loyalty. According to Aaker [51], brand associations are the foundation of purchasing decisions. In the educational field, brand associations refer to the information and associations made by the students about the academic service.

### 2.4. Satisfaction

Satisfaction is the extent to which a consumer experiences a pleasurable level of consumer satisfaction. It develops as a result of various interactions with the brand [66] and as a result of repeated positive reinforcements [67], having an essential role in developing strong and stable relationships between consumers and the brand [68]. Student satisfaction or dissatisfaction greatly influences staying or dropping out of college, influencing students’ retention or attrition [69]. Therefore, student satisfaction is an essential driver of student loyalty [60]. Students can be seen as the most important stakeholders for a university [70,71,72]. When they are satisfied, they choose other educational programs at the same university and recommend them to other people.

### 2.5. Trust

Trust refers to the ability and openness of an organization to honor its promises and fulfill its customer relationship obligations, being indispensable in forming and maintaining stable and collaborative relationships with customers [73,74]. Sirdeshmukh et al. [75] state that trust creates value because it provides relational benefits derived from the interaction between the organization and consumers, reducing uncertainty [76]. In the educational field, student trust develops through continuous personal experiences with the university. Based on the students’ personal experience with the university members and staff, students’ trust can represent confidence in the reliability and integrity of the university [27,77].

For the whole period 2009–2019, there was a trend of strengthening of “institutional trust” for Romania and Eastern Europe. The institutional trust as a concept explored in the relationship between students and the Bucharest University of Economic Studies is clearly part of the institutional trust in Romania. There are some measures of social trust under the Legatum Prosperity Index (LPI) or also under the World Values Survey. The LPI measures “institutional trust”, “interpersonal trust”, and “social networks” along with two other elements of “social capital”, while the WVS measures, among others, the general level of trust together with the level of trust in universities. The social capital pillar measures the strength of personal and social relationships, institutional trust, social norms, and civic participation in a country. In 2009, Romania ranked position 122 on the pillar of social capital, indicating a weak rank; in 2019, Romania advanced to position 116, indicating a small improvement (Legatum Prosperity Index, [78,79]). Additionally, based on the overall Legatum prosperity index, Romania advanced only one position, from 48th position in 2009 to 47th position in 2019. For the period 2020–2021, in the context of the crisis generated by COVID-19, there was a general trend of degradation of “institutional trust” and “interpersonal trust” in various countries, including Romania (Legatum Prosperity Index, [80]). In 2021, Romania ranks position 117th for the social capital pillar, marking a small decrease in the level of institutional trust, interpersonal trust, and social networks.

According to the 7th wave of the World Values Survey (2017–2021) [81], which reported the general level of trust of Romanians in 2018, a majority of 87% of the respondents mentioned the need of being very careful, and only 11.7% of them considered that most people can be trusted. Analyzing now the perceptions of Romanian students, 81.16% of them declared the need of being very careful and only 15.94% of them trusted most people. Regarding the perceived level of institutional trust in universities, the general opinion is that 56.48% of the respondents have quite a high level of trust in universities, while the specific opinion of students highlighted that 76.81% exhibited a high and very high level of trust in universities.

### 2.6. Commitment

Developing and maintaining long-term partnerships is the primary goal of relationship marketing. Commitment is essential in building a successful long-term relationship. It is defined as a belief that a relationship between partners must be maintained and continued with maximum effort [76] or as a psychological attachment to an organization [82]. Student commitment should be seen as perpetuating an ongoing sense of connection with the university or with its members [83]. According to Tinto (1975,1993) [84,85,86], student engagement is determined by their degree of integration, both academically (participation in university societies and committees) and socially (friends and acquaintances with fellow students).

Given the empirical results mentioned in the introduction, the next two research questions can be addressed:

1. What are the key factors influencing student loyalty?
2. What are the cause-and-effect relationships among the key factors influencing student loyalty?

Starting from the questions above, the following research hypotheses were formulated:

- **H1:** brand associations have a significant positive effect on student satisfaction.
- **H2:** perceived quality has a significant positive effect on student satisfaction.
- **H3:** student satisfaction has a significant positive effect on student trust.
- **H4:** student trust has a significant positive effect on student commitment.
- **H5:** student commitment has a significant positive effect on student loyalty.

The present research is quantitative, non-experimental, cross-sectional research, and the method we used is the survey. The research is much broader, including specific themes related to the marketing mix and brand equity, but this paper presents aspects related to the loyalty of students at a business university in Romania and its determinants. The instrument used in conducting this research is the questionnaire, which comprises a set of standardized and formalized questions. The population of this research is represented by all the students from ASE. In 2020, ASE will number 24,234 students, master’s and doctoral students (ANS, 2020), using as sampling layers the student gender distribution, age group distribution, and distribution of the main faculty followed by the student.

The sample included a total of 712 respondents, of which, after thorough checks of the questionnaires and the elimination of those with incomplete answers, 682 students remained. Thus, at a 95% confidence level, the margin of error is 4%. This research was conducted from January to March 2021, and the questionnaire was applied online through the Google Forms platform. There is very little research focusing on the effects of COVID-19 on student loyalty. To address this gap, we need to conduct studies during such a crisis to conclude if COVID-19 can influence student trust.

To achieve the research objectives, a questionnaire was developed based on two key marketing constructs, namely marketing mix and brand equity. The questionnaire in this paper consists of three main sections. The first section deals with the components of the marketing mix in the service industry, proposed by Booms and Bitner [87], namely product, price, promotion, distribution, people, physical facilities, process to which another component, called employability, was added, as it is considered an important factor in the selection of a university [88,89,90]. Each of the eight components contains four items, making a total of 32 items. The second section involves the dimensions of brand equity from the consumer perspective, namely brand awareness, brand loyalty, brand associations, and perceived quality, to which two other assets were added, namely satisfaction [91,92] and reputation [18,93,94]. Most dimensions contain four items, but brand awareness and reputation contain three items and satisfaction five. The second section contains 34 items. Likert scale with five response options (1—totally disagree and 5—totally agree) was used in the questionnaire.

The third section contains personal data, including gender, age, occupational status, academic program, year of study, major, form of funding, obtaining scholarships and what type of scholarships, plans for the future, interest in the field of study, and self-assessment of academic performance. The mentioned variables are structured as follows:

Gender: a dummy variable in which 1—female and 2—male.

Age: is a continuous variable quantifying the age of the respondent.

Occupational status: a polychotomous variable with the following variables 1—I work full time, 2—I work part time, 3—I’m not working, but I’m looking for a job, 4—I’m not working, and 5—I’m not looking for a job.

Academic program: a polychotomous variable with the following variables 1—bachelor program, 2—master’s program, and 3—doctoral program.

Year of study: a polychotomous variable with the following variables for the bachelor program 1—first year, 2—second year, and 3—third year; for the master program 1—first year, and 2—second year; for the doctoral program 1—first year, 2—second year, 3—third year, and 4—other years.

Major: a polychotomous variable with the following variables for the bachelor program 1—The Faculty of Business Administration (in Foreign Languages), 2—The Faculty of Administration and Public Management, 3—Bucharest Business School, 4—The Faculty of Business and Tourism, 5—The Faculty of Economic Cybernetics, Statistics and Informatics, 6—Faculty of Accounting and Management Information Systems, 7—Faculty of Law, 8—The Faculty of Agrifood and Environmental Economics, 9—The Faculty of Theoretical and Applied Economics, 10—The Faculty of Finance and Banking, 11—The Faculty of Management, 12—The Faculty of Marketing, 13—The Faculty of International Business and Economics; for the master program 1—The Faculty of Business Administration (in Foreign Languages), 2—The Faculty of Administration and Public Management, 3—Bucharest Business School, 4—The Faculty of Business and Tourism, 5—The Faculty of Economic Cybernetics, Statistics and Informatics, 6—Faculty of Accounting and Management Information Systems, 7—The Faculty of Agrifood and Environmental Economics, 8—The Faculty of Theoretical and Applied Economics, 9—The Faculty of Finance and Banking, 10—The Faculty of Management, 11—The Faculty of Marketing, 12—The Faculty of International Business and Economics; for the doctoral programs 1—Business Administration, 2—Economic Cybernetics and Statistics, 3—Accounting, 4—Economics, Economics and International Business, 5—Finance, 6—Economic Informatics, 7—Management, 8—Marketing, and 9—Law.

Form of funding: a polychotomous variable with the following variables 1—financing from the budget, with scholarship, 2—budget funding, no scholarship, 3—tax, and 4—other answers.

Obtaining scholarships: a dummy variable in which 1—yes and 2—no.

Type of scholarships: a polychotomous variable with the following variables 1—scholarships of excellence, 2—performance scholarships, 3—merit scholarships, 4—social scholarship, and 5—other answers.

Plans for the future: a polychotomous variable with the following variables 1—to get hired by state institutions, 2—to get hired by a private company, 3—to pursue an academic career, 4—to start my own business, and 5—other answers.

Interest in the field of study: a polychotomous variable with the following variables 1—to a very small extent, 2—to a small extent, 3—to some extent, 4—to a large extent, and 5—to a very large extent.

Self-assessment of academic performance: a polychotomous variable with the following variables 1—poor, 2—fair, 3—Satisfying, 4—good, and 5—very good.

The questionnaire is detailed in Appendix B.

As research methodology, within the analysis there have been developed two structural equation models (SEM) as follows: associations ≥ satisfaction ≥ trust ≥ commitment ≥ loyalty as well as perceived quality ≥ satisfaction ≥ trust ≥ commitment ≥ loyalty.

To investigate the degree of correlation within the set of items, Cronbach’s Alpha (c-alpha) was determined, which is the most common estimate of the internal consistency of items in a model. Thus, when the correlation is high, then it can be said that indicators can be used to build a composite indicator. An acceptable reliability threshold is 0.7 [95]. However, some authors use 0.75 or 0.80 as a limit value, while others consider it to be 0.6.

To evaluate the models and select the optimal model, the model accuracy was evaluated, including the statistical significance of the parameters, the value of the chi-square test that evaluates the model compared to the saturated model, root mean square error of approximation (RMSEA), residual standardized square mean root (SRMR), comparative fit index (CFI), Tucker–Lewis index (TLI) and coefficient of determination (CD) values, Akaike information criteria (AIC) values and Schwarz (SBC) [96].

The chi-square test evaluated the performance of the model compared to the saturated model, which fits perfectly with the covariances. To accept that the model fits, as well as the saturated model, the *p*-value must be 1. The RMSEA is the value of the square root approximation error, and the *p*-close is the probability that the RMSEA value is less than 0.05. The probability must be much closer to 1 in an optimal model. AIC and SBC criteria are used to compare models, with lower values indicating a better model. CFI and TLI are two indices whose values close to 1 indicate a good match. A value of 0 for SRMR indicates a perfect fit quality, while a value of less than 0.08 indicates a good fit. A value of the coefficient of determination close to 1 indicates a good quality of adjustment.

## 3. Results

### 3.1. The Profile of Romanian Students

From the total of 683 Romanian students interviewed, 23% are males, 77% are females, and the majority of them (60.6%) are between 20–22 years old. Almost 74% of the respondent students are enrolled in the bachelor’s program, 21% in the master’s program, and only 5% in doctoral programs at the Bucharest University of Economic Studies. The number of students enrolled in undergraduate programs is the highest, of which 35.84% are in year II, 33.66% are in year III, and 30.50% are in the first year. Regarding master’s programs, 54.17% of the responding students are in the first year, and 45.83% are in the second year. In the doctoral programs, 41.18% of the responding students are in the first year, 23.53% are in the second year, 20.59% are in the third year, and 14.71% are in the additional years.

Regarding the bachelor’s programs, there are no answers from students enrolled in the faculties of Accounting and Management Information Systems, the Faculty of Law, and the Bucharest Business School, and also none from students enrolled in master’s programs within the faculties of Accounting and Management Informatics and the Bucharest Business School. The distribution of students according to the specializations within the faculties of ASE is presented in Appendix C. Regarding the funding forms, the majority of the students, namely 42.46%, benefit from funding from the state budget without a scholarship, followed by students receiving state funding with a scholarship (31.19%) and students studying in a tax regime (26.35%).

Regarding the scholarships offered by ASE, the majority of responding students (61.64%) do not receive any scholarship, while 38.36% of students receive different types of scholarships, as follows: merit scholarship (70.99%), performance scholarship (21.37%), social scholarship (4.20%), and excellence scholarship (3.44%).

The occupational status of the students reveals the fact that most students (39.09%) are not currently working but are looking for a job, while 25.62% are not working and are not looking for a job. Moreover, 23.13% of the responding students work full time, and 8.64% are employed full time, while a small percentage, namely 3.51%, have their own business.

Regarding the career plan after graduation, 42.38% of the respondent students want to work in a private company, 29.18% intend to open their own business, 18.77% plan to work in various state institutions, and fewer than 9.68% of students want to pursue an academic career.

### 3.2. Highlighting the Main Determinants of Student of Loyalty

The descriptive statistics of all items included in the analysis are displayed in Table A1 from Appendix A, revealing that the first three components of trust exhibited the highest level of agreement: Trust 1: ASE is a trusted university, Trust 2: I always expect ASE to do the right thing, and Trust 3: ASE is an integral/honest/fair university.

The results were first examined using a correlation matrix (Table 1 and Table 2) between the main constructs: associations ≥ satisfaction ≥ trust ≥ commitment ≥ loyalty as well as perceived quality ≥ satisfaction ≥ trust ≥ commitment ≥ loyalty. The correlation matrices (Table 1 and Table 2) show that all correlation coefficients are significant at the 0.01 level.

Two structural equation models (SEM) that reveal the relationships among the latent variables were used to test for the validity of the measurements and to evaluate the usefulness of the models. For both models, confirmatory factor analysis (CFA) revealed an acceptable fit indices with all factor loadings ranging from 0.75 to 1 for the perceived quality model, and from 0.79 to 0.1 for the associations model. Reliability Cronbach’s alpha coefficients were above the minimum threshold of 0.70 recommended by Nunnally [89]: associations (0.942); perceived quality (0.87); satisfaction (0.947); commitment (0.949); loyalty (0.93); trust (0.925).

**Figure 2:**
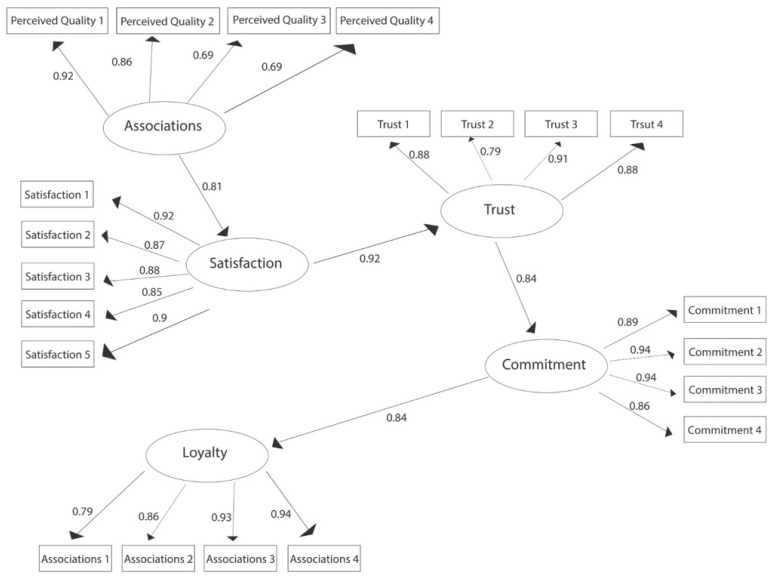
The structural model of the relationships between the components of brand equity (perceived quality, satisfaction, trust, brand commitment, brand loyalty) from the perspective of AES students.

The first structural model tested involves the relationships between perceived quality ≥ satisfaction ≥ trust ≥ commitment ≥ loyalty; the empirical results are presented in Figure 2.

Therefore, the empirical results validate hypotheses H2, H3, H4, and H5 in their entirety, meaning that there is a significant positive relationship between perceived quality, satisfaction, trust, commitment, and loyalty.

The empirical results of the first structural model analyzing the relationships between perceived quality ≥ satisfaction ≥ trust ≥ commitment ≥ loyalty revealed that, based on standardized path coefficients, satisfaction has the largest total effect on loyalty (0.92), followed by trust (0.84) and commitment (0.84), of which commitment shows a direct effect, while the other variables have only an indirect effect on loyalty. Satisfaction (0.92) and trust (0.84) show the strongest indirect effect on loyalty, while commitment (0.84) shows the strongest direct effect on loyalty. Together, perceived quality, satisfaction, trust, and commitment explain 92.4% of student loyalty, as derived from the CD of the structural equation model.

Regarding the testing of the hypotheses related to the first model, the results are shown in Table 3.

**Figure 3:**
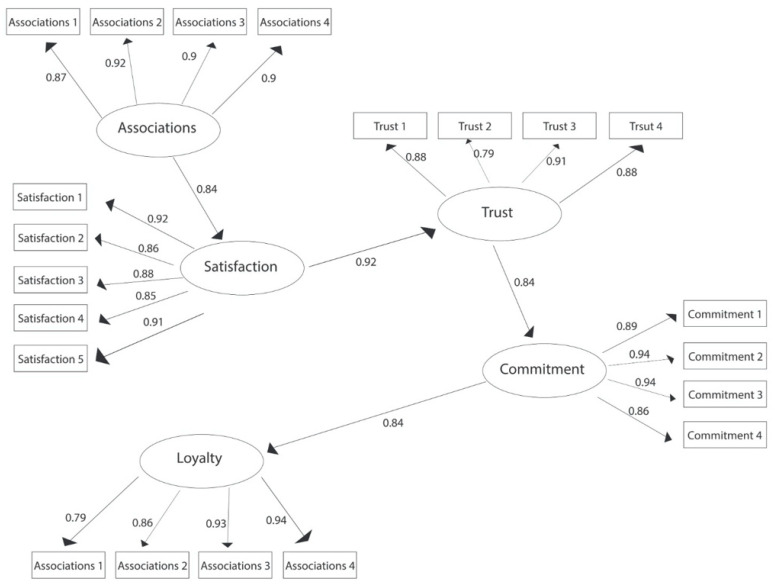
The structural model of the relationships between the components of brand equity (brand associations, satisfaction, trust, brand commitment, brand loyalty) from the perspective of AES students.

The empirical results of the second structural model examining the relationships between the associations of brand ≥ satisfaction ≥ trust ≥ commitment ≥ loyalty are shown in Figure 3.

As can be seen, satisfaction retains the largest indirect effect on loyalty (0.92), followed by trust (0.84) and brand associations (0.84). In contrast, commitment (0.84) shows the strongest direct effect on loyalty. Thus, of all these dimensions, only commitment has a direct effect on loyalty, while brand associations, satisfaction, and trust have an indirect effect on loyalty. Together, brand associations, satisfaction, trust, and commitment explain 95.2% of student loyalty, as shown by the CD of the structural equation model. 

Regarding the testing of the hypotheses formulated based on the second model, the results are shown in Table 4.

Therefore, the empirical results validate the hypotheses H1, H3, H4, and H5 in their entirety, meaning that there is a significant positive relationship between brand associations, satisfaction, trust, commitment, and loyalty.

The GFI and AGFI are above 0.95 and the RMSEA is under 0.05, indicating a good fit of the models to the data. The comparative fit index (CFI) and Tucker–Lewis index (TLI) are well above 0.90 registering 0.929 and 0.92 for the associations model, respectively, and 0.926 and 0.916 for the perceived quality model, respectively, indicating a good fit of the model to the data. Similarly, the other indices of comparative fit and parsimonious fit are above their recommended thresholds. The Hoelter’s (1983) critical N (which relates the adequacy of the sample size to the model) is well above 200, indicating that the samples are large enough to allow for an adequate fit to the model.

## 4. Discussion

The purpose of testing these two models was to explain student loyalty in higher education institutions by examining the key factors that influence loyalty. Perceived quality, brand associations, satisfaction, trust, commitment, and loyalty were reviewed in two models, which are comprehensive enough to explain loyalty. The empirical results showed that satisfaction has the greatest total effect on loyalty, followed by trust and commitment in both models. Commitment has the most significant direct impact on loyalty, mainly due to its direct and strong relationship with loyalty. The other factors have only indirect effects on loyalty and direct relationships in the following sequence: perceived quality to satisfaction, brand associations to satisfaction, satisfaction to trust, and trust to commitment. Therefore, in developing loyalty to the academic brand, university management must emphasize improving the quality of educational services, developing positive brand associations, and improving student satisfaction, trust, and commitment.

In many ways, these results are similar to those reported by Mazhar and Masood [97], who concluded that student satisfaction and perceived image influence loyalty, Chandra et al. [31] who revealed that loyalty is impacted by student satisfaction and the latter by service quality, and Annamdevula and Bellamkonda [30] who showed that student satisfaction has a mediator effect in the relationship between service quality and student loyalty. Moreover, similar results were also reported by Pham and Lai [98], who found out that affective commitment is an essential determinant of loyalty, this being the mediator in the relationship between satisfaction and loyalty, Purgailis and Zaksa [33], who concluded that loyalty is influenced by perceived quality through satisfaction, Perin et al. [34], who supported the idea according to which perceived quality influences student loyalty, but only indirectly through mediators such as trust and commitment, and, lastly, Henning-Thurau et al. [27], who concluded that loyalty is determined by service quality and brand commitment.

Therefore, in generating student brand loyalty, university management must first and foremost facilitate student satisfaction, which improves long-term relationships between them and the university. Enhancing student brand loyalty requires a deep understanding of the factors that influence satisfaction, the lack of which has negative consequences for both the university and the students [99,100,101]. Second, to build long-term relationships with all categories of stakeholders, the university must focus on developing their trust as part of relationships [29]. Lack of trust in the institution can negatively affect long-term relationships (Andaleeb, 1994, [29]). Third, because student commitment directly impacts loyalty, university management must make a considerable effort to develop it. Students’ level of commitment to an institution is closely related to their feelings, bonds, and identification with the institution. That is why student commitment should always be encouraged as loyal students to their universities can positively influence the quality of the educational process through their active participation.

In short, managers should increase student satisfaction towards the educational services to enhance students’ loyalty, which in turn maintains the survival rate of the university. Loyal students will always recommend the university to others and will keep future relational activities, such as returning when seeking future degrees. This stringent necessity of strengthening service quality and customer satisfaction is nowadays of paramount importance in such a competitive environment because students’ loyalty enhances higher education institutions’ sustainability and survival rate.

New research could explore service quality in the higher education system and particularly in the Bucharest University of Economic Studies. Likewise, service quality could be measured for each and every faculty in the university, comparing them with each other. Moreover, student loyalty could be measured in various contexts. For example, in specific careers and social groups. Other studies should focus on the loyalty of other stakeholders’ categories to the university, especially that of teachers and employers. The aim is to have more useful information and knowledge for the proper application of loyalty programs and strategies.

## 5. Conclusions

Student brand loyalty has become an essential strategic topic for higher educational institutions’ planning. The present results suggest no difference between students and consumers of other services because, over time, students have adopted client positions when interacting with the university [102]. In this regard, Pesch et al. [103] suggest that universities should adopt a customer orientation by focusing on the needs and wants of students. Education is a service like any other, and students, being customers, can best establish what they want from a university and evaluate the quality of education [104]. Therefore, managers should actively implement activities that build and maintain stable, lasting relationships by constantly providing high quality. For example, the institution’s environment should facilitate continuous learning for students. In addition, managers should ensure that services are fast and efficient, the academic staff should be professionals, and the administrative staff should be well mannered and give constant attention to the student’s needs and desires. Such measures will add to student satisfaction and loyalty to the university. Overall, the present study results prove the need to ensure and constantly increase service quality in higher educational institutions. In this way, positive brand associations are generated among students and increased satisfaction with the university, which will invariably affect student loyalty through trust and commitment. Student loyalty will always generate both a positive attitude and positive behavior towards the university and, therefore, a deep dedication to it, which can generate a substantial student involvement in the development and improvement of the institution.

In our view, to inspire more loyalty among students, any university should establish a strong relationship, which involves obligations and accountability, by behaving reasonably and keeping promises towards the students. To be respected, institutions need to treat students with fairness and respect. Moreover, institutions have to build a brand and project an identity that resonates with students’ wishes, interests, and expectations. Consequently, a university brand should strive to be positive, unique, and interactive. In building an effective academic brand, universities must first identify their target audience, know their needs, and then manage their mission, vision, and organizational culture accordingly. Being a brand that respects its audience shows a great understanding of them, which is what customer loyalty implies.

The research results indicate the importance of a strategic approach to brand and customer loyalty. Moreover, the results indicate that university management needs to focus on loyalty programs that correctly generate loyalty among the important stakeholder group: students. In such a competitive market as the education market, with different perceptions of the quality of educational services, loyalty, being a complex tool, can be an optimal way to achieve institutional goals and build solid and lasting relationships. This paper serves as a diagnostic tool for evaluating and improving loyalty among students with focused marketing efforts. The Bucharest University of Economic Studies could benefit from this research, integrating the approach into a new marketing strategy or improving the current marketing strategy by improving educational quality.

With regards to the main limitations of the study, first of all the present study extracted the sample from a single university in Romania. The final sample was indeed representative of the Bucharest University of Economic Studies student population, and the statistical modeling was robust. Still, future research should aim at reproducing the results using other student populations, for example, from other economic faculties in Romania, to have more conclusive and relevant results.

Secondly, the sample was extracted only from an economic university in Romania, representing only the student population within the Bucharest University of Economic Studies. Therefore, future research could take samples from several universities with different profiles in Romania to conduct comparative studies.

Finally, the present study is cross-sectional, which means that the results were recorded only once. A longitudinal study, in which research continues for a more extended period, using the same sample at each stage, would provide a much more concrete estimate of the measurement and performance of brand loyalty in higher education.

## Figures and Tables

**Table 1:** The correlation matrix between associations ≥ satisfaction ≥ trust ≥ commitment ≥ loyalty.

	Associations	Satisfaction	Trust	Loyalty	Commitment
associations	1				
satisfaction	0.877 *	1			
trust	0.812 *	0.9493 *	1		
loyalty	0.770 *	0.8386 *	0.8234 *	1	
commitment	0.7869 *	0.8725 *	0.8727 *	0.8735 *	1

Note: * means statistically significant at 1% significance level.

**Table 2:** The correlation matrix between perceived quality ≥ satisfaction ≥ trust ≥ commitment ≥ loyalty.

	Satisfaction	Trust	Commitment	Loyalty	Perceived Quality
Satisfaction	1				
Trust	0.9536 *	1			
Commitment	0.8704 *	0.8717 *	1		
Loyalty	0.8355 *	0.8224 *	0.8734 *	1	
Perceived Quality	0.8481 *	0.8097 *	0.7471 *	0.7269 *	1

Note: * means statistically significant at 1% significance level.

**Table 3:** Hypotheses testing in Rojas-Méndez et al.’s (2009) model explaining loyalty by perceived quality ≥ satisfaction ≥ trust ≥ commitment ≥ loyalty.

Hypotheses	Directions	*z*-Values	Standardized Path Coefficients (β)	Decision
H2	Perceived quality ≥ satisfaction	23.51 ***	0.81 ***	Validated
H3	Satisfaction ≥ trust	30.29 ***	0.92 ***	Validated
H4	Trust ≥ commitment	26.22 ***	0.84 ***	Validated
H5	Commitment ≥ loyalty	22.20 ***	0.84 ***	Validated

*** *p* < 0.01.

**Table 4:** Hypotheses testing in Rojas-Méndez et al.’s (2009) model, which explains loyalty through the associations of brand ≥ satisfaction ≥ trust ≥ commitment ≥ loyalty.

Hypotheses	Directions	*z*-Values	Standardized Path Coefficients (β)	Decision
H1	Brand associations ≥ satisfaction	24.28 ***	0.84 ***	Validated
H3	Satisfaction ≥ trust	29.84 ***	0.92 ***	Validated
H4	Trust ≥ commitment	26.24 ***	0.84 ***	Validated
H5	Commitment ≥ loyalty	22.20 ***	0.84 ***	Validated

*** *p* < 0.01.

## Data Availability

Data can be available upon request.

## Appendix A

**Table A1:** Descriptive statistics of variables.

Variable	Mean	Std. Dev.	Min	Max
satisfaction~1	3.786	1.121	1	5
satisfaction~2	3.782	1.117	1	5
satisfaction~3	3.770	1.167	1	5
satisfaction~4	3.870	1.221	1	5
satisfaction~5	3.603	1.197	1	5
Trust1	4.064	1.037	1	5
Trust2	3.930	1.205	1	5
Trust3	3.830	1.136	1	5
Trust4	3.679	1.164	1	5
Commitment1	3.580	1.264	1	5
Commitment2	3.640	1.268	1	5
Commitment3	3.624	1.260	1	5
Commitment4	3.731	1.263	1	5
Loyalty1	3.745	1.265	1	5
Loyalty2	3.660	1.319	1	5
Loyalty3	3.816	1.171	1	5
Loyalty4	3.801	1.188	1	5
Association~1	3.858	1.059	1	5
Association~2	3.791	1.116	1	5
Association~3	3.564	1.227	1	5
Association~4	3.635	1.207	1	5
Perceived_quality~1	3.376	1.193	1	5
Perceived_quality~2	3.447	1.234	1	5
Perceived_quality~3	3.606	1.194	1	5
Perceived_quality~4	2.909	1.454	1	5

## Appendix B. The Main Items of Brand Equity

	**Scale**	**Items**	**Adapted From:**
**1**	**Awareness**	I always recognize the ASE (Bucharest University of Economic Studies) logo	Pinar et al., 2014 [23]
		ASE is one of the first universities that comes to mind when I think of existing economic study programs in the country	
		I consider ASE to be a well-known university	
**2**	**Brand Associations**	ASE courses and seminars help me to acquire the skills necessary for my training as a specialist in the field studied	Aaker, 1991 [51] Keller, 2020 [105]
		ASE contributes significantly to my personal and professional development	
		ASE supports me in my personal and professional endeavour	
		ASE contributes to my career success	
**3**	**Perceived Quality**	The courses and seminars offered by ASE are always of a high quality	Aaker, 1991 [51] Jilllapalli and Jilllapalli, 2014 [106] Dennis et al., 2017 [107]
		University teachers are always well prepared	
		The physical facilities offered by ASE (amphitheaters, seminar rooms, laboratories, gyms) are always clean and well maintained	
		The administrative staff is always quick and eager to help the students	
**4**	**Trust**	ASE is a reliable university	Jilllapalli and Jilllapalli, 2014 Dennis et al., 2017 [107]
		I always wait for ASE to do the right thing	
		ASE is an integral /honest /fair university	
		ASE always keeps its promises	
**5**	**Satisfaction**	I am satisfied with the education I received at ASE	Bruner, 2009
		I am satisfied with the facilities offered to ASE students	
		I am satisfied with the way I was treated as a student at ASE	
		I think I did the right thing when I decided to choose ASE	
		I am satisfied with how ASE has prepared me for my career	
**6**	**Commitment**	I am very dedicated to this university	Jilllapalli and Jilllapalli, 2014 [106] Dennis et al., 2017 [107]
		This university is very important to me	
		I really care about this university.	
		I think this university is worth my effort to continue my studies here	
**7**	**Brand Loyalty**	I would always follow other educational programs (master, doctorate) within ASE	Helgesen and Nesset, 2007 [35]; Rojas-Méndez et al., 2009 [29]
		If I had to choose a university today, I would choose ASE	
		I often test positive for my acquaintances and /or friends	
		I would recommend studying at ASE to my relatives and friends	
**8**	**Reputation**	ASE has an excellent academic reputation	Plewa et al., 2015 [108]
		ASE has a good status in the education market	
		ASE is considered a prestigious institution	
**9**	**Brand Equity**	Compared to other universities, I would always prefer to study at ASE	Dennis et al., 2017 [107]
		If there was another university as good as ASE, I would also study at ASE	
		If another university were similar to ASE, I would also study at ASE	

## Appendix C. Respondent Students According to the Specializations within the Faculties Of ASE

**Faculties/Bachelor’s Degrees**		**Faculties/Master’s Degrees**		**Faculties/Doctoral Degrees**	
The Faculty of Business Administration (in Foreign Languages)	20.2%	Business Administration (in Foreign Languages)	10.4%	Business Administration	13.8%
The Faculty of Administration and Public Management	6.3%	Administration and Public Management	2.1%	Economic Cybernetics and Statistics	10.3%
The Faculty of Business and Tourism	4.5%	Business and Tourism	10.4%	Accounting	6.9%
The Faculty of Economic Cybernetics, Statistics and Informatics	19.4%	The Faculty of Economic Cybernetics, Statistics and Informatics	6.9%	Economics	17.2%
The Faculty of Theoretical and Applied Economics	7.3%	Theoretical and Applied Economics	5.6%	Economics and international affairs	13.8%
The Faculty of Finance and Banking	11.8%	Finance and Banking	9.7%	Finance	3.4%
The Faculty of Management	8.6%	Management	16%	Economic Informatics	3.4%
The Faculty of Marketing	7.3%	Marketing	13.9%	Management	13.8%
The Faculty of International Business and Economics	9.6%	International Business and Economics	8.3	Marketing	17.2%
The Faculty of Agrifood and Environmental Economics	5.1%	Agrifood and Environmental Economics	4.9%		
